# 5-grass-pollen SLIT effectiveness in seasonal allergic rhinitis: Impact of sensitization to subtropical grass pollen

**DOI:** 10.1016/j.waojou.2022.100632

**Published:** 2022-02-23

**Authors:** Sheryl A. van Nunen, Melanie B. Burk, Pamela K. Burton, Geoffrey Ford, Richard J. Harvey, Alexander Lozynsky, Elizabeth Pickford, Janet S. Rimmer, Joanne Smart, Michael F. Sutherland, Francis Thien, Heinrich C. Weber, Harry Zehnwirth, Ed Newbigin, Constance H. Katelaris

**Affiliations:** a Northern Clinical School, Sydney Medical School, Faculty of Medicine and Health, The University of Sydney, Sydney, Australia; b Department of Clinical Immunology and Allergy, Royal North Shore Hospital, Sydney, Australia; c Department of Medicine, Campbelltown Hospital, Sydney, Australia; d Essendon, Melbourne, Australia; e Rhinology and Skull Base Research Group, St Vincent's Centre for Applied Medical Research, University of New South Wales, Sydney, Australia; f Faculty of Medicine and Health Sciences, Macquarie University, Sydney, Australia; g Allergy Immunology Specialists, Westmead, Sydney, Australia; h Private Consultant Paediatric Allergy Practice, Castle Hill, Sydney, Australia; i Centre for Paediatrics, Faculty of Health Sciences, Macquarie University, Sydney, Australia; j Epworth HealthCare Richmond, Richmond, Australia; k Royal Children's Hospital, Melbourne, Australia; l The University of Melbourne, Melbourne, Australia; m Monash University, Melbourne, Australia; n Eastern Health, Melbourne, Australia; o Tasmanian Health Service-North West, Burnie, Australia; p Rural Clinical School, University of Tasmania, Burnie, Australia; q Ballarat Allergy Clinic, Ballarat, Australia; r School of BioSciences, The University of Melbourne, Melbourne, Australia; s Immunology/Allergy Unit, Department of Medicine, Campbelltown Hospital, Sydney, Australia; t School of Medicine, Western Sydney University, Sydney, Australia

**Keywords:** 5-grass pollen tablet, Allergen immunotherapy, Ryegrass, Allergic rhinitis, Polysensitization

## Abstract

**Background:**

Temperate grass (eg, ryegrass) pollen is a major driver of seasonal allergic rhinitis (SAR) and asthma risks, including thunderstorm asthma. Data for the effectiveness of temperate grass pollen allergen immunotherapy (AIT) in SAR patients from the southern hemisphere, who are frequently polysensitized to subtropical grass pollens, are limited. The 300 IR 5-grass pollen sublingual immunotherapy tablet (300 IR 5-grass SLIT) is known to be effective in polysensitized SAR patients with primary allergy to temperate grasses, however, the influence of polysensitization to subtropical grass pollen on treatment responses has yet to be specifically addressed. Key aims of this study were to measure patient treatment satisfaction during 300 IR 5-grass SLIT treatment and evaluate how polysensitization to subtropical grass pollens affects treatment responses.

**Methods:**

A prospective observational study was conducted in 63 patients (aged ≥5 years) in several temperate regions of Australia prescribed 300 IR 5-grass SLIT for SAR over 3 consecutive grass pollen seasons. Ambient levels of pollen were measured at representative sites. Patient treatment satisfaction was assessed using a QUARTIS questionnaire. Rhinoconjunctivitis Total Symptom Score (RTSS) and a Hodges-Lehmann Estimator analysis was performed to evaluate if polysensitization to subtropical grass pollen affected SAR symptom intensity changes during SLIT.

**Results:**

A diagnosis of ryegrass pollen allergy was nearly universal. There were 74.6% (47/63) polysensitized to subtropical and temperate grass pollens. There were 23.8% (15/63) monosensitized to temperate grass pollens. From the first pollen season, statistically significant improvements occurred in SAR symptoms compared with baseline in both monosensitized and polysensitized patients, particularly in those polysensitized (P = 0.0297). Improvements in SAR symptoms were sustained and similar in both groups in the second and third pollen seasons, reaching 70–85% improvement (P < 0.01). Polysensitized patients from both northerly and southerly temperate regions in Australia showed similar improvements. Grass pollen counts in both regions were consistently highest during springtime.

**Conclusions:**

300 IR 5-grass SLIT is effective in a real-life setting in SAR patients in the southern hemisphere with primary allergy to temperate grass pollen and predominantly springtime grass pollen exposures. Importantly, SLIT treatment effectiveness was irrespective of the patient's polysensitization status to subtropical grass pollens.

## Introduction

In temperate regions of Australia, and in similar climatic regions globally, pollens from temperate grasses (such as ryegrass) are major drivers of seasonal allergic rhinitis (SAR) and asthma risks, including epidemic thunderstorm asthma.^1^, ^2^, ^3^, ^4^

The effectiveness of temperate grass pollen allergen immunotherapy (AIT) has been well-characterized in SAR populations from the northern hemisphere, with evidence available from a number of large, double-blind, placebo-controlled, randomized trials for the short- and long-term treatment benefits of standardized AIT products.^5^ However, there have been comparatively few studies of the effectiveness of temperate grass pollen AIT treatments in SAR patients from the southern hemisphere, who are frequently polysensitized to both temperate and subtropical grass pollens.^1^^,^^2^

To date, one controlled study of grass pollen AIT effectiveness in Australian SAR patients has been performed, which was an open-label, controlled study conducted with 300 IR 5-grass pollen sublingual immunotherapy tablet (300 IR 5-grass SLIT), which contains a homologous mixture of temperate grass pollens including ryegrass pollen.^6^ This study by O'Hehir et al was conducted over 2–3 consecutive pollen seasons in Melbourne, Australia (2014–2016) and confirmed that 300 IR 5-grass SLIT is effective from the first pollen season in SAR patients with primary allergy to temperate grass (ryegrass) pollen.^6^ Notably, evidence was obtained that 300 IR 5-grass SLIT treatment also conferred protection against asthma exacerbations during an epidemic thunderstorm asthma event in November 2016,^6^ which is understood to have been triggered by acute exposure to respirable temperate grass pollen fragments enriched in Group 5 allergens, particularly ryegrass Lol p 5^3^^,^^4^^,^^7^).

Despite this body of evidence, important questions remain whether temperate grass pollen AIT can optimally benefit SAR patients with primary allergy to temperate grass pollen where these patients display polysensitization to other pollen allergens. In the northern hemisphere this question has been determined by analyses of large double-blind, placebo-controlled studies and real-world studies conducted in the United States and Europe, which have established that 300 IR 5-grass SLIT is effective in SAR patients irrespective of their polysensitization status to non-homologous allergens such as tree and ragweed pollen.^8^, ^9^, ^10^, ^11^ However, these studies did not evaluate outcomes in patients from the southern hemisphere and, importantly, they did not specifically examine how polysensitization to subtropical grass pollen may influence 300 IR 5-grass SLIT effectiveness. Addressing this specific area of uncertainty is of considerable practical importance for informing optimal approaches to AIT for SAR patients in temperate regions of Australia, and similar regions globally, where temperate grass pollen is a major driver of SAR disease burdens and risks and polysensitization to subtropical grass pollen is prevalent.^1^, ^2^, ^3^, ^4^

In the present study, patient treatment satisfaction during treatment with 300 IR 5-grass SLIT over 3 consecutive pollen seasons was evaluated in a real-life setting in SAR patients with primary allergy to temperate grass pollen in temperate regions of Australia. In a pre-specified subgroup analysis, the impact of patient polysensitization status to subtropical grass pollen on changes in SAR symptom intensity during 300 IR 5-grass SLIT treatment was evaluated.

## Methods

### Study design

**Fig. 1:**
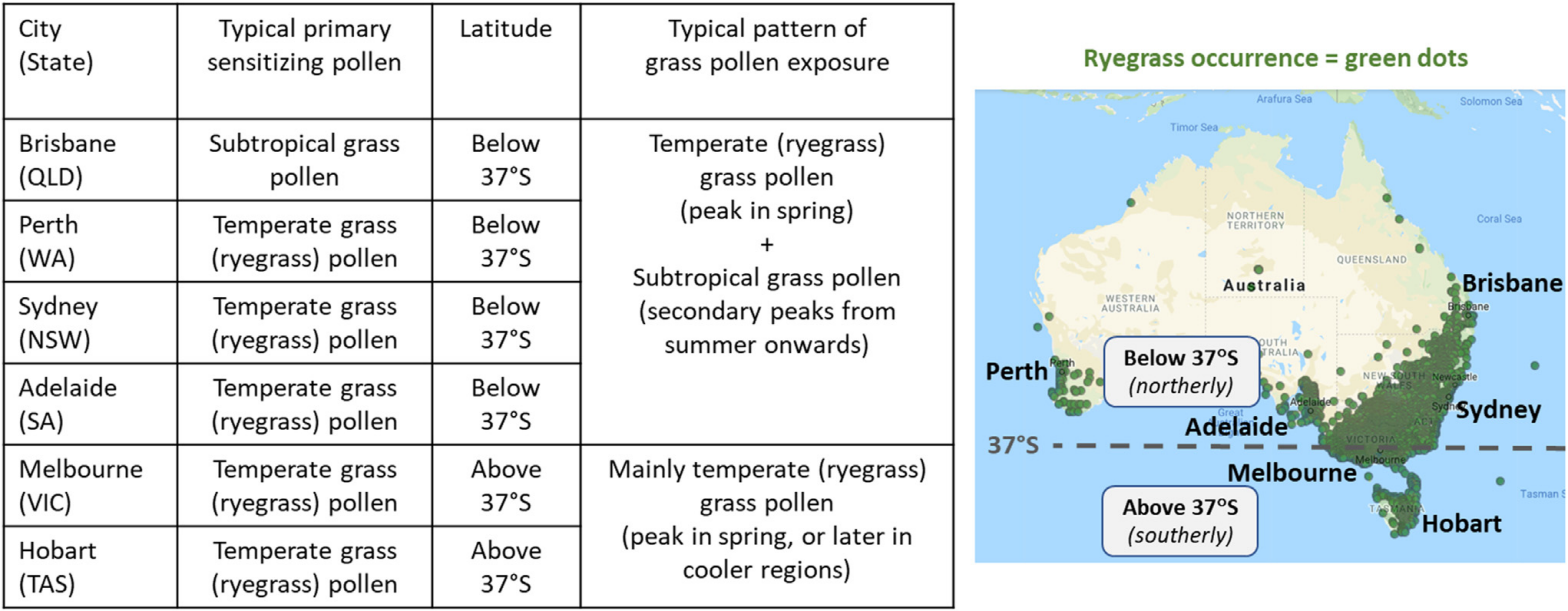
**Typical biogeographical patterns of grass pollen exposure**^2^^,^^18^^,^^20^**and sensitization**^2^^,^^18^^,^^30^**in Australia.** QLD = Queensland; WA = Western Australia; NSW = New South Wales; SA = South Australia; VIC = Victoria; TAS = Tasmania. Ryegrass occurrence data is from the Living Atlas of Australia (http://www.ala.org.au/).

This was a prospective, open-label, uncontrolled, multi-centre, non-interventional study in usual practice settings (private consultant allergists’ practices and public hospital allergy clinics) conducted in eastern Australian states (New South Wales, Australian Capital Territory, Victoria, and Tasmania) between May 2013 and February 2016 (see map in Fig. 1). The study protocol was approved prior to commencement of the study. Study participants were required to give written informed consent.

**Fig. 2:**
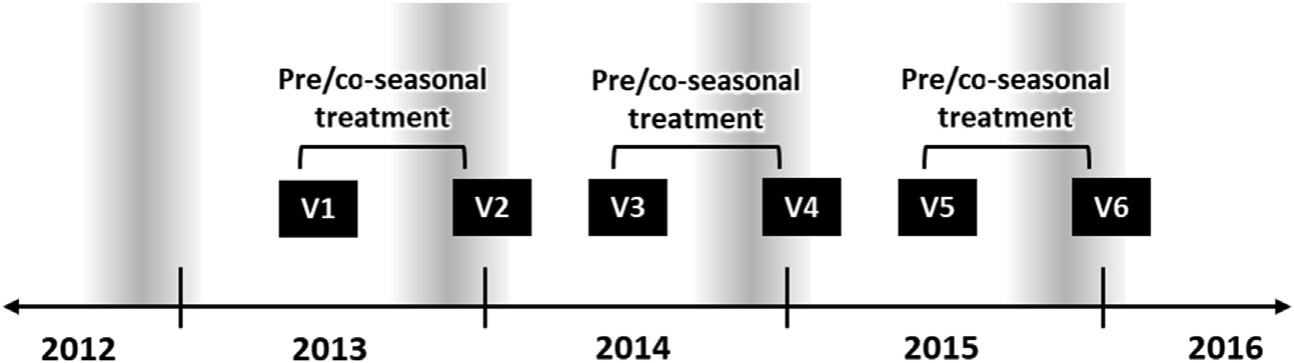
**Study design.** The pollen seasons are indicated by the shaded bars. Study visits (V1–V6) were scheduled before (V1, V3, V5) and after (V2, V4, V6) each grass pollen season. Data were documented retrospectively for the immediately preceding pollen season at V1, V2, V4, and V6, with "baseline" values for the 2012–2013 pollen season documented at V1.

The observation phase was conducted during 6 Visits (V1 – V6) over 3 consecutive pollen seasons, commencing prior to the 2013–2014 pollen season. In each pollen season, a pre-pollen season visit was conducted between May 15 and August 15 (V1, V3, and V5) and a post-pollen season visit was conducted between December 1 and February 28 of the following year (V2, V4, and V6) (Fig. 2). Demographic data, skin prick tests (and/or serum specific IgE tests) and medical/allergy history (including assessment of the relationships between sensitizations and clinical symptoms, as well as SAR and asthma severity according to ARIA (Allergic Rhinitis and Its Impact on Asthma)^12^ and GINA (Global Initiative for Asthma) classifications^13^ were documented at V1. AR symptom intensities experienced during the pollen season were documented retrospectively at V1, V2, V4, and V6 for symptoms experienced over the immediately preceding pollen season, with values documented at V1 representing "baseline" values for the 2012–2013 pollen season. AR symptom intensities experienced outside the pollen season were documented retrospectively at V3 and V5 (for the periods preceding the 2014–2015 and 2015–2016 pollen seasons) (Fig. 2, Fig. 4). Retrospective documentation of AR symptoms at each study visit was supplemented by the use of written patient diaries and the quality of data collection was assessed by a clinical trial nurse and/or treating consultant, as well as by an independent study monitor.

**Fig. 3:**
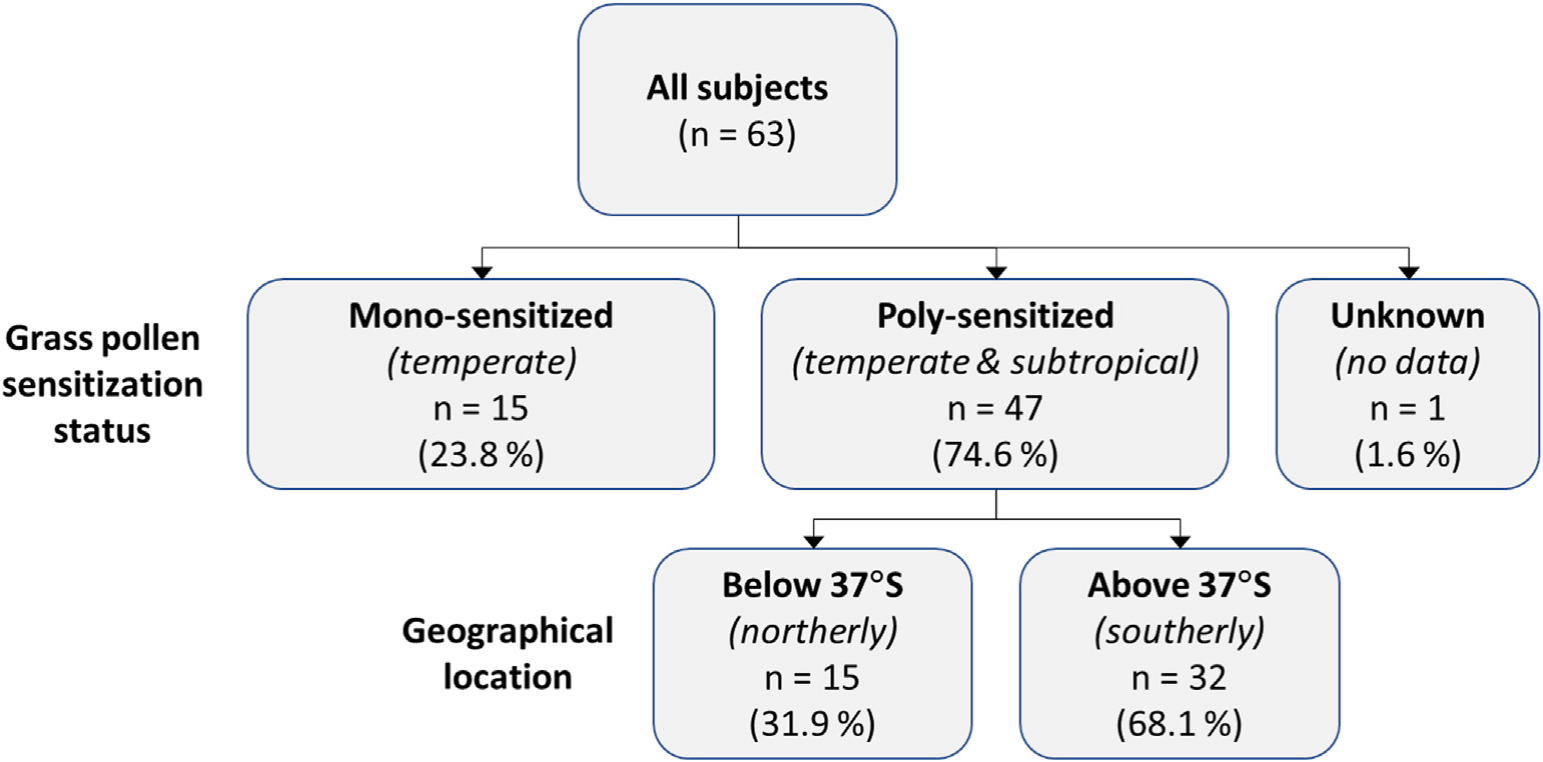
**Flow chart of study subjects stratified by patient subgroups, according to grass pollen sensitization status and geographical location.** Monosensitized = sensitized to temperate grasses only. Polysensitized = sensitized to temperate and subtropical grass pollens. Below (south of) 37°S = patients located in more northerly temperate regions. Above (north of) 37°S = patients located in more southerly temperate regions.

**Fig. 4:**
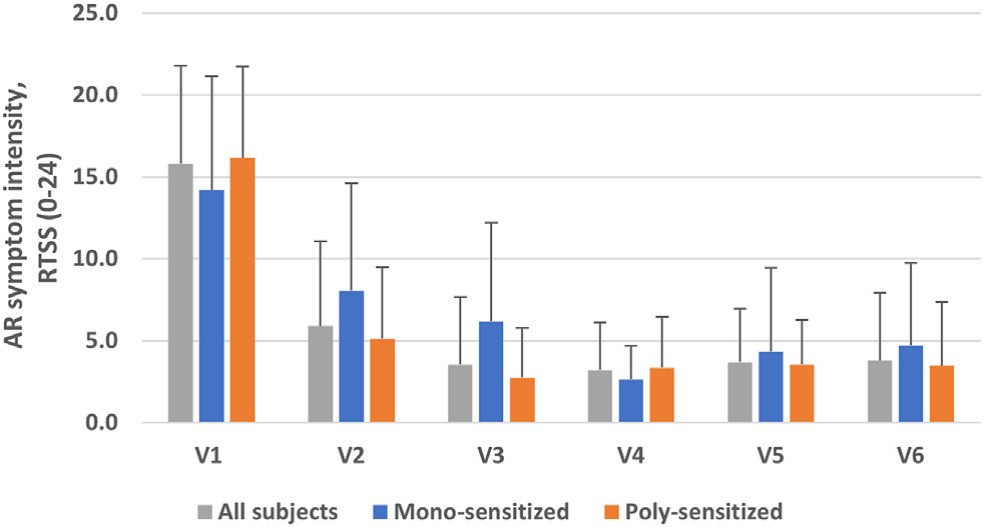
**RTSS (0–24) in sub-groups stratified according to grass pollen sensitization status (see**Table 2 for statistical comparisons between sub-groups). Monosensitized = sensitized to temperate grasses only. Polysensitized = sensitized to temperate and subtropical grass pollens. V1, V2, V4, and V6 = RTSS values for periods during consecutive pollen seasons (V1 = 2012–2013 pollen season; V2 = 2013–2014 pollen season; V4 = 2014–2015 pollen season; V6 = 2015–2016 pollen season). V3 and V5 = RTSS values for periods between consecutive pollen seasons (V3 = before the 2014–2015 pollen season; V5 = before the 2015–2016 pollen season). Values are mean ± SD.

### 5-grass SLIT treatment

ORALAIR® is a 300 IR 5-grass SLIT tablet (300 IR 5-grass SLIT), which contains pollen extracts from ryegrass (*Lolium perenne* L.), orchard/cocksfoot grass (*Dactylis glomerata* L.), meadow grass (*Poa pratensis* L.), sweet vernal grass (*Anthoxanthum odoratum* L.), and Timothy (*Phleum pratense* L.). The biological activity of the tablet is expressed in the manufacturer-specific unit “Index of Reactivity” (IR). Patients were prescribed daily sublingual treatment with 300 IR 5-grass SLIT (ORALAIR®) on a pre/co-seasonal treatment schedule according to the Australian Product Information, with treatment initiation occurring at V1 (Fig. 2). It is recommended treatment be initiated approximately 4 months prior to the expected start of the pollen season and is composed of 2 phases: the three-day dose escalation phase (Day 1: 1 × 100 IR tablet, Day 2: 2 × 100 IR tablets, Day 3: 1 × 300 IR tablet) is followed by a maintenance phase until the end of the pollen season with 1 × 300 IR tablet per day. For tablet intake, the tablets are kept under the tongue until complete dissolution (at least 2 min) and are swallowed subsequently.

### Patient selection

Patients (aged ≥5 years) with SAR due to grass pollen allergy who had been given 300 IR 5-grass SLIT during the usual course of their treatment (according to the Australian Product Information) were eligible for recruitment. Thus, inclusion criteria were grass pollen AR with or without conjunctivitis in adults, adolescents, and children (above the age of 5) with clinically relevant symptoms, known to be confirmed by a positive grass pollen skin prick test and/or a positive titre of serum specific IgE to the grass pollen with usual AIT contraindications applied. According to these entry criteria (supported by recorded clinical history and sensitizations; see below), all study participants had primary allergy to temperate grass pollen.

Patients were recruited by allergy specialists at 12 study sites responsible for the management of allergic patients located in temperate regions of eastern Australia (New South Wales, Australian Capital Territory, Victoria [encompassing Melbourne/southern Victoria] and Tasmania (see map in Fig. 1).

Patient grass pollen sensitization status was classified as follows: (i) monosensitized = documented sensitization to at least one temperate grass pollen (ryegrass, orchard/cocksfoot grass, meadow grass, sweet vernal grass, Timothy); (ii) polysensitized = documented sensitizations to at least one temperate grass pollen (as above) and at least one subtropical grass pollen (Bermuda grass [*Cynodon dactylon*], Johnson grass [*Sorghum halepense*]); and (iii) unknown = no documented grass pollen sensitizations.

Patient geographic location (see map in Fig. 1) was characterized as follows: (i) above 37°S (south of 37°S) = patients receiving treatment in more southerly temperate regions (Victoria and Tasmania); and (ii) below 37°S (north of 37°S) = patients located in more northerly temperate regions (New South Wales and the Australian Capital Territory).

### Data collection methods

The primary study objective was to investigate in a real-life setting patient satisfaction during the last 3 months of the pollen season (regarding symptom improvement, ease of use, frequency of dosage) following 300 IR 5-grass SLIT treatment using a modified QUARTIS questionnaire (a validated self-report questionnaire that is used to assess patient-reported outcomes in patients with AR who are treated with SLIT)^16^^,^^17^ over a full treatment course of 3 pollen seasons. Patient-reported satisfaction was documented retrospectively at V2, V4, and V6 on 3 measures: (i) symptom improvement ("much more than/more/just as/less than/much less than, I hoped"); (ii) ease of use ("very satisfied/satisfied/neither satisfied or dissatisfied/dissatisfied/very dissatisfied"); and (iii) frequency of dosing ("very satisfied/satisfied/neither satisfied or dissatisfied/dissatisfied/very dissatisfied").

A pre-specified secondary objective was to determine the impact of polysensitization status to subtropical grasses upon changes in AR symptom intensity during SLIT over 3 consecutive pollen seasons. Changes in AR symptom intensity were indexed by change versus baseline analysis of the Rhinoconjunctivitis Total Symptom Score (RTSS) (Table 2). The RTSS was calculated as the sum of individual symptom evaluations (sneezing, itchy nose, runny nose, blocked nose, eyes itching, eyes watering) and converted to a numerical score (0–24) based on the following scale, which was also used to index the intensity of asthma symptoms: Not at all bothered = 0; Slightly bothered = 1; Quite bothered = 2; Very bothered = 3; Extremely bothered = 4.

**Table 1:** Number of patients commencing and completing treatment in each of the 3 pollen seasons

Pollen season	Commenced pre-seasonal treatment N (%)	Completed co-seasonal treatment N (%)
2013–2014	63 (100.0)	56 (88.9)
2014–2015	46 (73.0)	39 (61.9)
2015–2016	30 (47.6)	29 (46.0)

**Table 2:** Impact of grass pollen sensitization status on RTSS (0–24) during the pollen season (V2, V4, and V6): Change versus baseline (V1) analysis and statistical comparisons between sub-groups. *Monosensitized = sensitized to temperate grasses only. Polysensitized = sensitized to temperate and subtropical grass pollens. The baseline RTSS was similar for polysensitized patients (baseline RTSS = 16.16; n = 45) and monosensitized patients (baseline RTSS = 14.20; n = 15). Hodges-Lehmann Estimator analysis was used to estimate differences between median RTSS values for the mono- and polysensitized groups. Paired Student T-Test was used for other statistical comparisons.*

Visit	Statistics	All subjects	Mono sensitized	Poly sensitized
V2	N	54	15	39
	**Mean RTSS change vs baseline**	**−9.9**	**−6.1**	**−11.4**
	(SD)	(7.1)	(8.8)	(5.8)
	Paired Student t-test		p = 0.0171	p < 0.0001
	**Difference vs monosensitized**			**−6**
	Hodges-Lehmann Estimator			p = 0.0297
V4	N	37	8	29
	**Mean RTSS change vs baseline**	**−12.4**	**−12.0**	**−12.5**
	(SD)	(5.4)	(7.3)	(5.3)
	Paired Student t-test		p = 0.0074	p < 0.0001
	**Difference vs monosensitized**			**−1**
	Hodges-Lehmann Estimator			P = 0.8398
V6	N	27	7	20
	**Mean RTSS change vs baseline**	**−11.5**	**−12.0**	**−11.4**
	(SD)	(6.0)	(7.9)	(5.5)
	Paired Student t-test		p = 0.0069	p < 0.0001
	**Difference vs monosensitized**			**1**
	Hodges-Lehmann Estimator			p = 0.8048

A post-hoc analysis was performed to determine the impact of patient geographical location on the outcome of the secondary analysis, comparing changes in the RTSS during SLIT in polysensitized patients from northerly temperate regions (below 37°S [north of 37°S]) vs southerly temperate regions (above 37°S [south of 37°S]) (Fig. 1) (Table 3). The rationale for differentiating between these geographic regions is that data from pollen monitoring studies at sites across Australia and New Zealand indicate that the contribution of subtropical grasses to pollen exposures is greater in temperate regions below (north of 37°S) than in more southerly temperate regions above (south of 37°S)^18^ (Fig. 1).

**Table 3:** Impact of geographical location on RTSS (0–24) during pollen seasons: Change versus baseline analysis. *Polysensitized = sensitized to temperate and subtropical grass pollens. Below/above 37°S = patient geographical location. The baseline RTSS was similar for polysensitized AR patients from regions below <37°S (baseline RTSS = 15.00; n = 15) and above <37°S (baseline TRSS = 16.73; n = 30)*

	Statistics	Polysensitized	
		Below 37°S	Above 37°S
V2	N	12	27
	**Mean RTSS change vs baseline**	**−12.5**	**−10.9**
	(SD)	(5.4)	(5.9)
	Paired Student t-test	p > 0.05	p > 0.05
V4	N	10	19
	**Mean RTSS change vs baseline**	**−11.5**	**−13.0**
	(SD)	(5.1)	(4.5)
	Paired Student t-test	p > 0.05	p > 0.05
V6	N	8	12
	**Mean RTSS change vs baseline**	**−9.5**	**−12.6**
	(SD)	(5.0)	(5.6)
	Paired Student t-test	p > 0.05	p > 0.05

Daily grass and total pollen concentrations were measured during 4 consecutive pollen seasons (commencing with the 2012–2013 pollen season) using a Burkard volumetric trap at sites in Melbourne, Victoria (data collection from October 1 to December 31 each pollen season), and Sydney, New South Wales (data collection from September 1 to January 31 of the following year in each pollen season).^14^^,^^15^5-grass SLIT treatment compliance (V2, V4, and V6), use of medication to alleviate symptoms (for AR and asthma; all visits) and other AIT (all visits) were also documented. To evaluate safety, any occurrence of an adverse event was documented and classified according to the Medical Dictionary for Regulatory Activities (MedDRA; https://www.meddra.org/).

### Statistical methods

Data were analysed from all patients who participated. There was no imputation of missing values and change versus baseline analyses of the RTSS were only performed for subjects with documented baseline data at V1. Differences in change versus baseline of the RTSS between subgroups were analysed using a Hodges-Lehmann Estimator analysis (Wilcoxon rank sum test, normal approximation, two-sided p-value).

## Results

### Study population

**Fig. 5:**
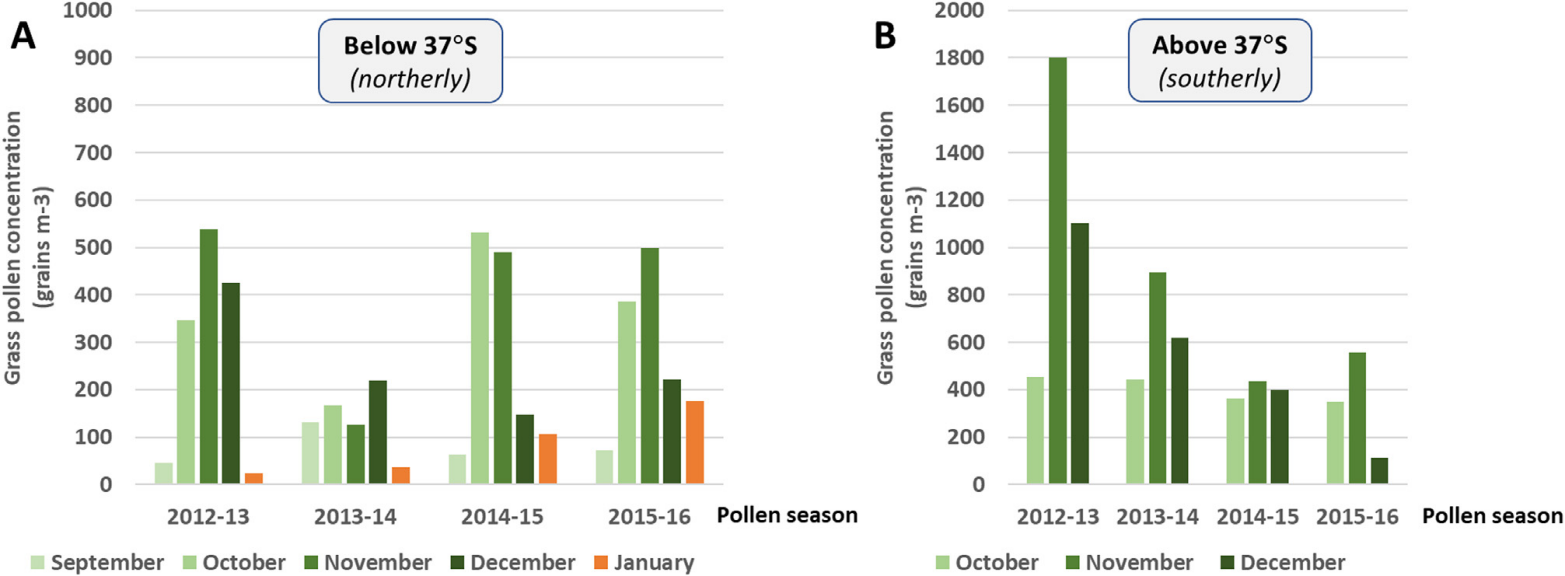
**Seasonal distributions of grass pollen.** Cumulative monthly grass pollen counts during relevant pollen seasons were calculated from measurements at two representative sites in temperate regions (A) below 37°S (Sydney, New South Wales) and (B) above 37°S (Melbourne, Victoria).

A total of 63 patients with primary allergy to temperate grass pollen (34 male, 29 female), with a mean age of 24.6 ± 16.0 (SD) years (35 children, 28 adults) were included in the study. Dropouts between V1 – V2 numbered 7 (11.1%) and similar decreases between consecutive visits were observed throughout the study period, both during and after completion of co-seasonal SLIT treatment courses (Table 1). Over the three year study period, 10 patients (15.9%) reported discontinuing prematurely, 6 of these in relation to adverse events. Post-pollen-season study visits at the completion of co-seasonal SLIT treatment (V2, V4, and V6) were scheduled during summertime, after peak springtime grass pollen exposures (see Study Design). Due to Australia's location in the southern hemisphere, grass pollen seasons in temperate regions extend over 2 calendar years, with springtime grass pollen exposures generally peaking around November and with summertime grass pollen exposures continuing through January of the following year^1^^,^^18^ (Fig. 5).

At baseline, the severity of AR and asthma symptoms were classified according to ARIA (n = 63) and GINA (n = 62) criteria respectively. A large majority of subjects had persistent (n = 49; 77.8%) and moderate-to-severe rhinitis (n = 58; 92.1%). A majority of subjects had asthma (n = 33; 53.2%); of these, about one-half had their asthma partially controlled (n = 15), about one-half had their asthma controlled (n = 17) and 1 subject had uncontrolled asthma.

At baseline, all subjects with documented sensitizations to grass pollen (n = 62) were sensitized to temperate grass pollen. One patient had no documented grass pollen sensitizations (incomplete data), but was judged to meet the study inclusion criteria and (therefore) have primary allergy to temperate grass pollen. Ryegrass pollen was the most common temperate grass pollen sensitization (n = 59/63; 93.7%) and, where the relevant allergy history was documented (n = 56), ryegrass pollen allergy was near universal (n = 55/56; 98.2%). Bermuda grass pollen was the most common subtropical grass pollen sensitization (n = 39/63, 61.9%) and, where the relevant allergy history was documented (n = 38), Bermuda grass pollen allergy was common (n = 23/38; 60.5%). Of non-pollen allergens, house dust mite sensitization was most common (n = 33, 52.4%) and where the relevant allergy history was documented, house dust mite allergy was common (n = 17/33; 51.5%).

Stratification of patient subgroups according to polysensitization status and geographical location is summarized in Fig. 3. There were 74.6% of subjects (n = 47) polysensitized to temperate grass pollen and subtropical grass pollens. Of these polysensitized patients (n = 15), 31.9% were located in northerly temperate regions below 37°S (north of 37°S) (New South Wales and Australian Capital Territory) whilst 68.1% were located in southerly temperate regions above 37°S (south of 37°S) (Victoria and Tasmania).

Mean duration of AR symptoms prior to 5-grass SLIT treatment was 12.2 (SD: 9.1) years (n = 63). The majority of subjects were AIT-naïve (n = 55). Eight subjects switched from an alternative AIT treatment (SCIT, n = 4; SLIT drops, n = 4). Concomitant AIT use was low: 5-grass SLIT was the only grass pollen AIT treatment used during the study period from V1 – V5; 1 subject was on Bermuda grass SLIT at V6; at selected visits, 1–2 subjects were on HDM AIT (V2, n = 2; V4, n = 1; V5, n = 1). Compliance with 5-grass SLIT was good, with a large majority of patients (96.4–100%) reporting their daily usage as "most of the time" or "always" at the end of each pollen season (V2, V4, and V6), the remainder reporting daily usage as "sometimes".

### Patient treatment satisfaction

Subject treatment satisfaction was queried following 300 IR 5-grass SLIT treatment at the end of each pollen season (V2, V4, and V6). At each of these visits, a large majority of subjects experienced symptom improvements that exceeded or met their expectations ("much more than/more than/just as, I hoped": 82.1–89.1%) and there were low levels of dissatisfaction with ease of use ("dissatisfied/very dissatisfied": 3.6–7.1%) and frequency of dosing ("dissatisfied/very dissatisfied": 3.6–5.4%).

### Changes in AR symptom intensity

In the overall study population, the baseline RTSS (0–24) was 15.8 (SD: 6.0) (n = 61). The baseline RTSS in subgroups stratified according to grass pollen sensitization status was similar: monosensitized to temperate grass pollen = 14.2 (SD: 6.9) (n = 15); polysensitized to temperate and subtropical grass pollens = 16.2 (SD: 5.6) (n = 45) (Fig. 4). In all groups, the RTSS was lower at all subsequent study visits following 300 IR 5-grass SLIT treatment (Fig. 4), as were all component AR symptom scores (sneezing, itchy nose, runny nose, blocked nose, eyes itching, eyes watering).

Change versus baseline analysis of the RTSS showed that the intensity of AR symptoms during the pollen season was significantly decreased in all groups (all subjects, monosensitized and polysensitized) from the first pollen season following 5-grass SLIT treatment (V2), with these decreases maintained during the second and third pollen seasons (V4 and V6) (Table 2). At V2, AR symptom reductions in the polysensitized group were significantly greater compared to the monosensitized group (p = 0.0297; Hodges-Lehmann estimator analysis) (Table 2). At V4 and V6, the monosensitized group showed further AR symptom reductions, minimizing differences to the polysensitized group, with both groups reaching 75–80% improvement vs baseline at V6 (p < 0.01; Table 2).

AR symptom reductions in polysensitized patients from geographical regions below (north of) and above (south of) 37°S were very similar, despite potential differences in the level of subtropical grass pollen exposure between these groups (Table 3; see Fig. 1).

### Changes in the intensity of asthma symptoms

At baseline (V1) 33 patients reported having asthma and 19 patients reported being quite/very/extremely bothered by their asthma symptoms (representing 32% of those that reported asthma symptoms at this study visit). From V2 onwards, no more than 6 patients reported these levels of asthma symptoms (representing 2–14% of those that reported asthma symptoms at each study visit).

### Pollen measurements

The seasonal distributions of grass pollen measured at representative sites in the northerly temperate region below 37°S (north of 37°S) (Sydney) and in the southerly temperate region above 37°S (south of 37°S) (Melbourne) were analysed for relevant pollen seasons. Cumulative grass pollen counts were calculated on a monthly basis to clarify the timing of peak grass pollen levels in each region (Fig. 5).

In both regions, grass pollen levels were consistently highest during springtime, around November (Fig. 5), which is the expected timing of peak exposures to temperate grass (ryegrass) pollen in temperate regions of Australia.^18^ As previously reported by Silver et al,^14^ grass pollen exposures also peaked during springtime (around November), in the Australian Capital Territory during an overlapping study period (2014–2016).

In the region below 37°S (north of 37°S) (Sydney), data were captured for an extended period (until January 31), ensuring that measurements encompassed the expected timing of peak summertime exposures to subtropical grass pollen, around January.^18^ In each pollen season, cumulative grass pollen counts during January (summertime) were considerably lower than during November (springtime) and represented a comparatively minor fraction of cumulative seasonal grass pollen counts (2–13%) (Fig. 5A). Although data were not captured in January in Melbourne, the overall distribution of pollen counts indicates a trend for similarly low grass pollen counts during this month (Fig. 5B).

### Usage of medication for AR and asthma symptoms

At baseline, regular use of medication for symptoms of AR was common, with "daily" or "often" usage reported at V1 as follows: oral antihistamines (71%), antihistamine nasal spray (27%), antihistamine eye drops (21%), corticosteroid nasal spray (35%); a smaller proportion of patients reported "daily" or "often" usage of medication for symptoms of asthma: β-sympathomimetics (13%), inhaled corticosteroid (22%) and oral corticosteroid (2%). Over the course of the study, there were consistent trends for decreased usage of medication for symptoms of AR and asthma during the pollen season (methodological considerations precluded change versus baseline and statistical analyses; see Methods). "Daily" or "often" usage of oral antihistamines during the pollen season fell dramatically and remained below 7% from the first pollen season (V2). "Daily" or "often" usage of intranasal corticosteroids during the pollen season fell to 18% during the first pollen season and to <4% during subsequent pollen seasons. Regular usage of medication for symptoms of asthma during the pollen season rose during the first pollen season, however, during subsequent pollen seasons "daily" or "often" usage fell to below 7% for each class of asthma treatment.

### Safety

A total of 62 adverse events were reported by 23 subjects. Thus 36.5% of subjects (n = 23) reported an event of any type, with gastrointestinal disorders (n = 17; 27.0%) and respiratory, thoracic, and mediastinal disorders (n = 11; 17.5%) being the most prevalent system organ classes reported.

## Discussion

Evaluating how a patient's polysensitization status should influence approaches to AIT treatment is a fundamental clinical challenge. This challenge can be addressed with a considerable degree of certainty when (i) a patient's clinical history can clearly identify the major causal allergen(s), (ii) a well-characterized, standardized AIT treatment is available that can appropriately target these causal allergen(s), and (iii) there is evidence that this AIT treatment can be highly effective in populations with similar characteristics to the patient under consideration. A particular practical challenge for the management of SAR in the southern hemisphere, compared to the northern hemisphere, has been the relative lack of evidence from local studies for the grass pollen AIT effectiveness in SAR patient populations who present with polysensitization to locally common aeroallergens, such as subtropical grass pollen.

In temperate regions of Australia, where the majority of the population resides, SAR patients being considered for AIT therapy commonly present with a clinical history indicating primary allergy to temperate grass pollens (that is, their most troublesome allergy symptoms occur predominantly during springtime) but also display sensitization to subtropical grass pollen (see Fig. 1). Although the effectiveness of 300 IR 5-grass SLIT has been confirmed in Australian SAR patients,^6^ the present study provides an important additional advance by evaluating, for the first time, how polysensitization to subtropical grass pollen impacts upon the effectiveness of 300 IR 5-grass SLIT in patients with primary allergy to temperate grass (ryegrass) pollen.

The main finding of this prospective observational study — of methodological relevance according to the recent proposed hierarchy of Real-World Evidence (RWE)^19^ — is that 300 IR 5-grass SLIT treatment in Australian SAR patients (both children and adults) with primary allergy to temperate grass pollen from several temperate regions of Australia (New South Wales, Australian Capital Territory, Victoria, and Tasmania), and including patients with polysensitization to subtropical grass pollen, was associated with high levels of patient-reported treatment satisfaction.

Consistent with these high levels of treatment satisfaction, there were marked decreases in the intensity of SAR symptoms versus baseline during 300 IR 5-grass SLIT treatment from the first pollen season, which were sustained through 3 consecutive pollen seasons, in patients with and without polysensitization to subtropical grass pollen. In the first pollen season, SAR symptom improvements in patients with polysensitization to subtropical grass pollen even slightly exceeded those in patients with monosensitization to temperate grass pollen. Furthermore, these improvements occurred in polysensitized patients regardless of their geographical location, including in more northerly (above 37°S) temperate regions where the potential for exposures to subtropical grass pollen is highest (see Fig. 1).

Overall, these novel findings reinforce and extend findings from large, double-blind, placebo-controlled trials and real-world studies conducted in the northern hemisphere, which have established that 300 IR 5-grass SLIT is effective in SAR patients with primary allergy to temperate grass pollen from the first pollen season and that polysensitized patients achieve at least the same magnitude of treatment efficacy as monosensitized patients.^8^, ^9^, ^10^, ^11^ The high levels of treatment satisfaction observed during 300 IR 5-grass SLIT treatment in Australian SAR patients likewise reinforce the outcomes of large observational studies in Europe, where 300 IR 5-grass SLIT treatment was also shown to be associated with high levels of treatment satisfaction.^17^

The observed seasonal patterns of grass pollen counts may assist in understanding why 300 IR 5-grass SLIT treatment was similarly effective in all patient subgroups analysed, stratified by polysensitization status to subtropical grass pollen and by geographical location (Fig. 3). A key finding was that grass pollen counts were consistently highest during springtime (November), when peak exposures to temperate grass pollens are expected, and were relatively low during summertime (January), when peak exposures to subtropical grass pollen are expected^3^^,^^18^^,^^20^ (see Fig. 1). This raises the possibility that spring-flowering temperate grasses (rather than summer-flowering subtropical grasses) may have been a predominant cause of SAR symptoms during this study. Although pollen-specific analyses are needed to confirm this hypothesis, the particular importance of springtime exposures to temperate grass pollens in driving the burdens and risks of SAR in temperate regions of Australia is supported by other data: for example, recent studies have identified that springtime peaks in temperate grass pollen in temperate regions correlate with springtime peaks in self-reported SAR symptoms and hospital admissions due to seasonal asthma, including during epidemic thunderstorm asthma.^3^^,^^4^^,^^14^^,^^21^^,^^22^

The major limitation of this study is the small number of patients studied. Minor limitations include the instances of incomplete patient data, a recognised limitation inherent in the conduct of real-world studies.^23^ Not all subjects in the monosensitized group were tested, or had data, to confirm a lack of sensitization to subtropical grasses. The effect of any bias introduced by this limitation, however, would be to minimize differences between the subgroups and would not favour increased efficacy outcomes in the poly versus the monosensitized groups, as was observed. Furthermore, the reported rate of polysensitization to subtropical grass pollen in the whole study cohort (75%) is consistent with values in the literature for SAR populations in similar geographic locations (72.4%).^24^

With regard to pollen measurements, an identified limitation is that their predictive value for SAR symptoms is sensitive to patient proximity to the measurement site.^25^ Therefore, whilst the observed data are useful to broadly indicate the likely timing of peak grass pollen exposures across the study population (for example, springtime versus summertime), more detailed inferences regarding relationships between cumulative seasonal grass pollen counts and treatment responses to 300 IR 5-grass SLIT are necessarily limited as measurements were performed only at 2 representative sites amongst 12 study sites. Importantly, baseline AR symptom scores and AR symptom score reductions were very similar in patients from different geographic regions (Table 3).

As SAR patients with primary allergy to temperate grass pollens are known to be at particular risk of life-threatening asthma exacerbations during epidemic thunderstorm asthma events (even for those without previously diagnosed asthma),^3^^,^^4^^,^^26^ international AR management guidelines specifically highlight the importance of considering AIT for this at-risk population, even when AR is controlled by pharmacotherapy.^27^ Within this context, considering SAR patient suitability for well-characterized, standardized and clinically-proven temperate grass pollen AIT treatments such as 300 IR 5-grass SLIT assumes added importance in clinical practice.

Evaluating patient suitability for different AIT treatments involves assessing the relative importance of different allergens in driving the individual patient's disease and assessing which available AIT treatments can most effectively target the major causal allergen(s). As highlighted above, a possible explanation for why treatment responses to 300 IR 5-grass SLIT in patients from the temperate regions examined in this study were uniformly high, regardless of patient sensitization status or geographical location, is that SAR symptoms were largely driven by springtime exposures to temperate grass pollen, with subtropical grass pollen only playing a relatively minor role in driving symptoms, even in northerly temperate regions below 37°S (north of 37°S) (cf. Fig. 5). Further studies are needed to investigate this possibility; if correct, in SAR patients subject to predominantly springtime grass pollen exposures, there may only be limited scope for improving clinical outcomes by additionally targeting AIT to subtropical grass pollen. Furthermore, adopting this approach may involve consideration of less well-characterized AIT treatments, which carries risk: whilst available AIT treatments containing mixtures of temperate and subtropical grass pollen have theoretical benefits, they have yet to be studied in relevant patient populations as no controlled clinical studies have been conducted to date. As such, there is no certainty that these treatments can match the proven effectiveness of 300 IR 5-grass SLIT in addressing disease burdens and risks due to temperate grass pollen allergy, both in the short- and long-term.^6^^,^^8^

Given the prevalence of SAR patients globally with similar characteristics and pollen exposures to those examined in this study, the findings of this study should have broad relevance globally for informing optimal approaches to AIT. For example, primary allergy to temperate grass pollen and polysensitization to subtropical grass pollen is likely be a common clinical presentation in temperate regions of South Australia, Western Australia, New Zealand, and North America (see Fig. 1). The findings from this study are likely to be relevant to temperate regions of North America, where SAR patients from temperate climate zones typically display primary sensitization to temperate grass pollen,^28^ temperate grasses are an important source of grass pollen during springtime,^29^ and subtropical grasses contribute to secondary pollen peaks during summertime.^28^, ^29^, ^30^

In summary, this real-life study of children and adults with SAR and primary allergy to temperate grass (ryegrass) pollen located in temperate regions of Australia showed that 300 IR 5-grass SLIT treatment was associated with high levels of treatment satisfaction and was associated with high levels of SAR symptom improvements, irrespective of patient polysensitization status to subtropical grass pollen or geographical location. These findings should have broad relevance globally and should increase certainty about the characteristics of SAR patients who can be suitable for this well-characterized, standardized and clinically-proven temperate grass pollen AIT treatment.

## Abbreviations

AIT, Allergen ImmunoTherapy; AR, Allergic Rhinitis; ARIA, Allergic Rhinitis and its Impact on Asthma; GINA, Global Initiative for Asthma; HDM, house dust mite; IR, Index of Reactivity; MedDRA, Medical Dictionary for Regulatory Activities; QUARTIS, Questionnaire sur l’Allergie Respiratoire Traitée par Immunothérapie Sublinguale; RWE, Real-World Evidence; RTSS, Rhinoconjunctivitis Total Symptom Score; SAR, Seasonal Allergic Rhinitis; SLIT, Sublingual Immunotherapy; V, Visit.

## Funding

Stallergenes Greer provided assistance with research protocol preparation for submission, costs of submission to the HRECs, statistical analysis and artwork for publication. The cost of 300IR 5-grass SLIT treatment was funded by the subjects themselves (real-life, post-marketing study). Our clinical research nurse was funded by the NSLHD (Northern Sydney Local Health District) Staff Specialists Trust Fund #2 of Prof. Sheryl van Nunen.

## Authors’ consent for publication

I confirm that each of the authors has reviewed this paper in its submitted form and approved submission for publication of this paper to the World Allergy Organization Journal.

## Author contributions

SvN contributed to the design of the study, prepared and supervised the Ethics Committee submissions, recruited patients for the study, supervised the study overall, reviewed the analyses of the data and wrote the manuscript with the participation of all authors.

MBB supervised patients throughout the study, collated study data, liaised with the sponsor regarding the study's progress and reviewed the data analyses and the manuscript.

CHK, ED, PKB performed, analysed and presented the pollen counting, reviewed the clinical data analyses and the manuscript.

GF, RJH, AL, EP, JSR, JS, MFS, FT, HCW and HZ recruited patients for the study, supervised data collation from their patients, reviewed the data analyses and reviewed and contributed to the manuscript preparation.

## Availability of data and materials

Any data and materials pertaining to this study not included in the paper are available from the corresponding author upon application.

## Ethics approval

The study protocol was approved prior to commencement of the study by the Bellberry Human Research Ethics Committee (2012-04-737) (https://bellberry.com.au/) and by the Northern Sydney Local Health District (NSLHD) Human Research and Ethics Committee (LNR/12/HAWKE/68 1202-061 M). Study participants were required to give written informed consent.

## Declaration of competing interest

SvN, MBB, PKB, GF, AL, EP, JSR, MFS, FT, HCW, HZ, EN and CHK, have no conflict of interest to declare.

JS declares membership of Advisory Boards for Takeda and CSL. RJH declares consultancies/Advisory Board membership of Medtronic, Novartis, GSK and Meda Pharmaceuticals. Research grant funding received from Glaxo-Smith-Kline. Speakers' bureau for Glaxo-Smith-Kline, AstraZeneca, Meda Pharmaceuticals and Seqirus.

## References

[1] Davies J.M., Berman D., Beggs P.J. (2021). Global climate change and pollen aeroallergens: a southern hemisphere perspective. Immunol Allergy Clin. North Am..

[2] Kailaivasan T.H., Timbrell V.L., Solley G. (2020). Biogeographical variation in specific IgE recognition of temperate and subtropical grass pollen allergens in allergic rhinitis patients. Clin Transl Immunol.

[3] Davies J.M. (2017). https://eprints.qut.edu.au/110102/.

[4] Lee J., Kronborg C., O'Hehir R.E., Hew M. (2017). Who's at risk of thunderstorm asthma? The ryegrass pollen trifecta and lessons learnt from the Melbourne thunderstorm epidemic. Respir Med.

[5] Roberts G., Pfaar O., Akdis C.A. (2018). EAACI guidelines on allergen immunotherapy: allergic rhinoconjunctivitis. Allergy.

[6] O'Hehir R.E., Varese N.P., Deckert K. (2018). Epidemic thunderstorm asthma protection with five-grass pollen tablet sublingual immunotherapy: a clinical trial. Am J Respir Crit Care Med.

[7] Hew M., Lee J., Varese N. (2020). Epidemic thunderstorm asthma susceptibility from sensitization to ryegrass (Lolium perenne) pollen and major allergen Lol p 5. Allergy.

[8] Didier A., Wahn U., Horak F., Cox L.S. (2014). Five-grass-pollen sublingual immunotherapy tablet for the treatment of grass-pollen-induced allergic rhinoconjunctivitis: 5 years of experience. Expet Rev Clin Immunol.

[9] Malling H.J., Montagut A., Melac M. (2009). Efficacy and safety of 5-grass pollen sublingual immunotherapy tablets in patients with different clinical profiles of allergic rhinoconjunctivitis. Clin Exp Allergy.

[10] Shah-Hosseini K., Krudewig E.M., Hadler M., Karagiannis E., Mösges R. (2017). Management of grass pollen allergy with 5-grass pollen tablet: results of a 2-year real-life study. Adv Ther.

[11] Pepper A.N., Calderón M.A., Casale T.B. (2017). Sublingual immunotherapy for the polyallergic patient. J Allergy Clin Immunol Pract.

[12] Brożek J.L., Bousquet J., Agache I. (2017). Allergic rhinitis and its impact on asthma (ARIA) guidelines-2016 revision. J Allergy Clin Immunol.

[13] Virchow J., Bufe A., Seitzberg D., Bateman E. (2011). Global initiative for asthma classification of asthma control derived from the asthma control questionnaire data. Allergy.

[14] Silver J.D., Spriggs K., Haberle S. (2020). Crowd-sourced allergic rhinitis symptom data: the influence of environmental and demographic factors. Sci Total Environ.

[15] Shrestha S.K., Katelaris C., Dharmage S.C. (2018). High ambient levels of grass, weed and other pollen are associated with asthma admissions in children and adolescents: a large 5-year case-crossover study. Clin Exp Allergy.

[16] Reig A.R., Casas C.P., Fernández D.G. (2021). A retrospective nationwide non-interventional study of an aqueous sublingual immunotherapy formulation administered with a 200-μL dosing pump. Drugs - Real World Outcomes.

[17] Schäfer U., Kienle-Gogolok A., Hadler M. (2017). Treatment satisfaction during sublingual immunotherapy with a five-grass pollen tablet for allergic rhinoconjunctivitis: a prospective, non-interventional study. Drugs Real-World Outcomes.

[18] Medek D.E., Beggs P.J., Erbas B. (2016). Regional and seasonal variation in airborne grass pollen levels between cities of Australia and New Zealand. Aerobiologia.

[19] Paoletti G., DiBona D., Chu D.K. (2021). Allergen immunotherapy: the growing role of observational and randomized trial "Real-World Evidence. Allergy.

[20] Derrick E. (1962). Relative importance of various plants in causation of hay fever and asthma in Australia. Med J Aust.

[21] Silver J.D., Sutherland M.F., Johnston F.H. (2018). Seasonal asthma in Melbourne, Australia, and some observations on the occurrence of thunderstorm asthma and its predictability. PLoS One.

[22] Hayden T.J., Muscatello D.J. (2011). Increased presentations to emergency departments for asthma associated with rye grass pollen season in inland NSW. NSW Publ Health Bull.

[23] Nazha B., Yang J.C., Owonikoko T.K. (2021). Benefits and limitations of real-world evidence: lessons from *EGFR* mutation-positive non-small-cell lung cancer. Future Oncol.

[24] Kam A.W., Tong W.W., Christensen J.M. (2016). Microgeographic factors and patterns of aeroallergen sensitisation. Med J Aust.

[25] Silver J.D., Spriggs K., Haberle S.G. (2020). Using crowd-sourced allergic rhinitis symptom data to improve grass pollen forecasts and predict individual symptoms. Sci Total Environ.

[26] Cockcroft D.W. (2018). Epidemic thunderstorm asthma. Lancet Planet Health.

[27] Bousquet J., Pfaar O., Togias A. (2019). ARIA Care pathways for allergen immunotherapy. Allergy.

[28] Davies J.M. (2014). Grass pollen allergens globally: the contribution of subtropical grasses to burden of allergic respiratory diseases. Clin Exp Allergy.

[29] Lo F., Bitz C.M., Battisti D.S., Hess J.J. (2019). Pollen calendars and maps of allergenic pollen in North America. Aerobiologia.

[30] Davies J.M., Li H., Green M. (2012). Subtropical grass pollen allergens are important for allergic respiratory diseases in subtropical regions. Clin Transl Allergy.

